# Unraveling multimodality of digital health records by comparing mortality trajectories of diagnoses of diseases from over 12 million patients

**DOI:** 10.1371/journal.pone.0314993

**Published:** 2025-02-04

**Authors:** Hyojung Paik

**Affiliations:** 1 Department of Data & High Performance Computing Science, University of Science and Technology (UST), Yuseong-gu, Daejeon, South Korea; 2 Division of National Supercomputing, Center for Biomedical Computing, Korea Institute of Science and Technology Information (KISTI), Yuseong-gu, Daejeon, South Korea; Nanyang Technological University, SINGAPORE

## Abstract

Understanding the multimodality of digital health data, including the scope of death records, is essential for adequate data acquisition to build a research framework for the health sciences. In this study, I leveraged the diverse healthcare records of over 12 million patients to reconstitute mortality trajectories that navigate the sequence of disease processes shared among patients from initial presentation through interim conditions that ultimately terminate in fatal outcomes. I conducted a comprehensive analysis of longitudinal discharge records for 10.4 million patients from US hospitals, utilizing the US State Inpatient Data (USSID) including 290,253 records of deaths in clinics. I also scrutinized the cross-sectional records of Korea from the billing reviews, specifically the National Inpatients Set of Korea (NISK), encompassing 2.1 million patients. By tracing the diagnostic timelines of patients diagnosed with significant comorbid diseases (False Discovery Rate (FDR) <0.1), I built mortality trajectories, mapping the temporal progression of disease diagnoses resulting in death. My trajectory model rewired 705 significant mortality trajectories across both datasets (USSID and NISK). The presented mortality trajectories successfully recapitulated established patterns of mortality for each country, while also revealing different trajectories leading to death, influenced by the modality of data. For example, viral hepatitis, a known predisposing feature of liver cancer in Asia, was observed to initiate in younger Koreans. Interestingly, owing to the collection of hospital records, the modeled mortality trajectories derived from the USSID converged towards sepsis. Although a substantial sequence of diagnostic processes is shared between USSID and NISK, the multimodality of these two datasets highlights different diagnoses preceded by fatal outcomes. Unraveling mortality patterns is feasible with an appropriate understanding of the multimodality of digital health data.

## Introduction

Adequate identification and risk stratification of individuals [1] are pivotal aspects of clinical decision-making for public health [2–4]. However, the modality (i.e. structure or scope) of health data are heterogeneous based on the purpose of data generation. For example, owing to the preference of home death, substantial death records are missed in the hospital records of US [5]. While understanding the multimodality of digital health data, including the scope of death records is essential, attempts to unravel the multimodality of digital health data using an identical approach are rarely suggested. Epidemiological studies using medical records and questionnaires can compare the prognostic values of risk factors for disease co-occurrence [6, 7] and mortality [8]. Many previous studies have focused on determining the risk factors for mortality [9] or disease comorbidity [10, 11]. Extensive analyses of comprehensive health registry data have facilitated data-driven approaches to understanding illness trajectories in Denmark [12]. Enormous efforts, such as the Global Burden of Disease, have revealed the existence of conserved yet partially diverse trends in illness [13] and causes of death, which vary by nationality, gender, and age [14]. While a previous attempt presented a comparison of treatment pathways following the transition of original data via the Common Data Model [15], the impact of source-dependent observations remains unclear. By applying the identical analytics onto distinct scope of health data, such as administrative hospital record and insurance reviews, the comparison of results would show the contribution of health data modality for in-depth analysis. Therefore, here, I compare multimodal digital health datasets by leveraging identical approaches.

In previous attempts, I repurposed population-wide administrative healthcare records covering different sources, including hospital records and insurance reviews. Using Directed Acyclic Graph (DAG) modeling, we identified unexpected risks of schizophrenia [16] and cancer [17] in the US. Similarly, the convergence of genomic evidence, based on the DAG approach, and digital health data accelerates the discovery of biological knowledge, such as a novel pleiotropic variant underlying disease comorbidity [18]. Overall, the utilization of digital health data derived from various sources and systemic reviews demonstrates the methodological robustness of the DAG.

The Healthcare Cost and Utilization Project (HCUP) [19] has released the State Inpatient Dataset (SID) as an encountered-level longitudinal record. For the US State Inpatients Dataset (USSID), we utilized the SID for California, encompassing over 10.4 million hospitalized patients from all community hospitals in California, spanning 20 years of hospital records. As an independent set of the USSID, we also obtained the National Inpatients Set of Korea (NISK) [20] from the Health Insurance Review & Assessment Service (HIRA) of Korea. This dataset comprises a cross-section of billing reviews for one year, covering over 2.1 million patients from all hospitals in Korea. Data from administrative hospital records and billing reviews were publicly available for application research. From our previous attempts, we utilized scaled digital health records from different data sources to validate our hypothesis [17, 18]. For example, in one of our previous works, we only used USSID to identify unexpected comorbidity risk without mortality consideration based on its modality [16]. Therefore, an understanding of data modality builds adequate interpretation of health outcome-related studies.

Utilizing these datasets, I conducted a systematic investigation to evaluate mortality trajectories, specifically the patterns of disease preceding death shared among patients. Because of the inherently distinct resolutions of disease coverage captured by each dataset, US data (USSID) were employed as the primary set to model the longitudinal trajectory between disease and death. However, I utilized the Korean dataset (NISK) to demonstrate the robustness and validity of the DAG method for analyzing administrative billing records. By examining the deadliest mortality trajectories, I identified corresponding known and significant clinical burdens in the US (myocardial infarction) and Korea (liver cancer). Within the well-portrayed mortality patterns of the two countries, my trajectory model revealed data modality-biased death patterns. Understanding the hetero modality of digital health data is a fundamental requirement for employing our trajectory model.

## Methods

### Population-wide administrative healthcare records in two different countries

**Fig 1:**
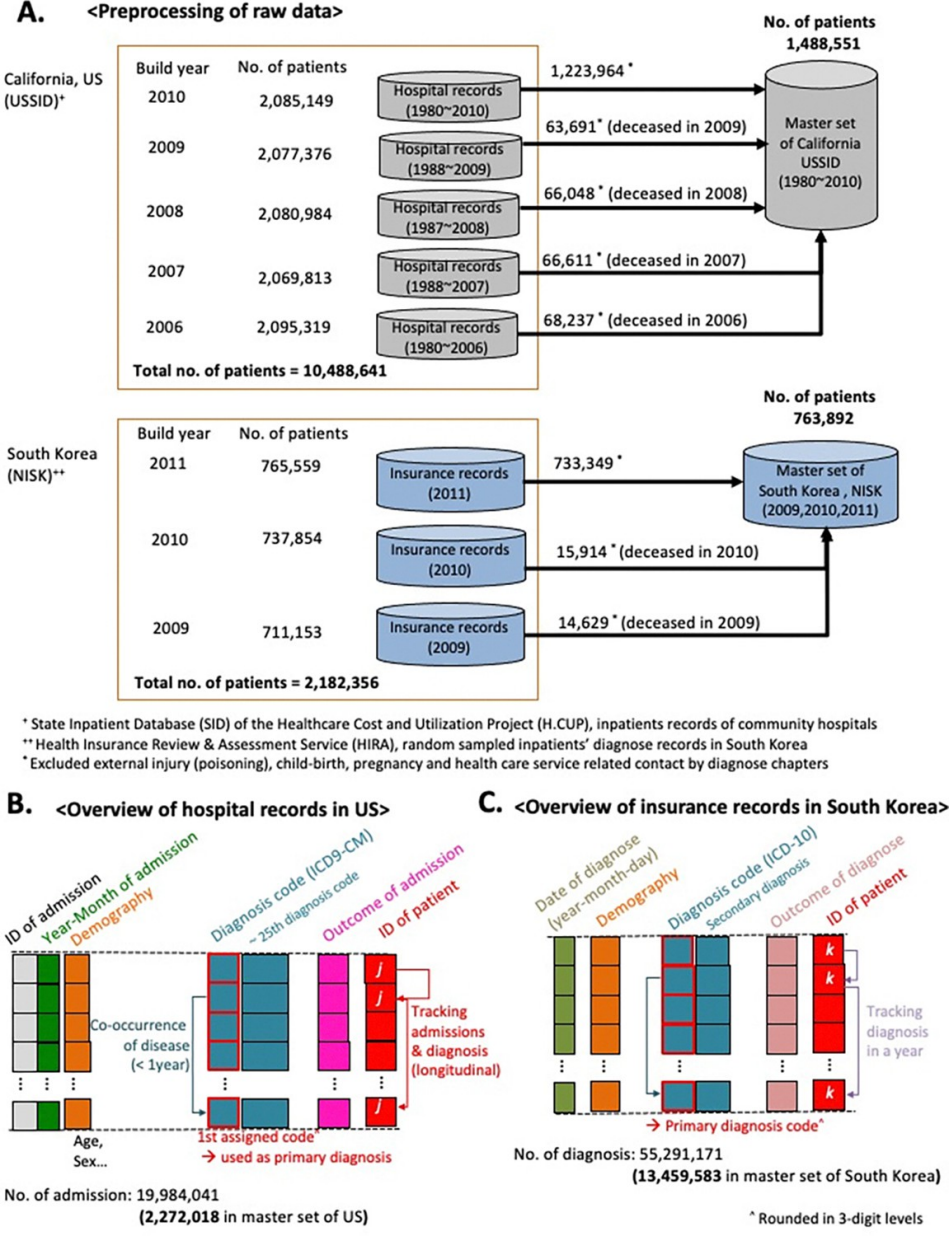
Overview of data and analysis. (A) Preprocessing of data and overview of analysis methods. We prepared the primary data set for the US by combining five data sets of the USSID, which are released annually (2006~2010). Similarly, the National Inpatients Set of Korea (NISK) was utilized to integrate three sets of the released version from 2009 to 2011. NISK covers all of the outpatient records who have admission records in a year. (B-C) Overall modality of utilized USSID (B) and NISK (C).

The analysis utilized two distinct datasets. The USSID: This dataset comprises the California set of the SID of the HCUP (Healthcare Cost and Utilization Project); and the NISK: This another dataset was obtained from the HIRA (Health Insurance Review and Assessment) (details in Data Availability). Based on the International Classification of Disease, Ninth Revision, Clinical Modification (ICD-9-CM) codes, I excluded all records of non-disease conditions, such as injuries or healthcare contact. Due to the annual compilation and release of USSID datasets, I independently selected records of deceased patients in the early generated dataset and subsequently merged them with later data releases to minimize the redundancy of records. For NISK, I also excluded non-disease conditions based on the diagnosis codes consisting of International Classification of Disease, Tenth Revision (ICD-10) (Fig 1A).

To minimize confounding impact from racial composition, I used USSID of California to compare with NISK. Both of NISK and USSID are healthcare records in administrative level, such as diagnosis codes, admission records, and outcome of discharge. The admission and discharge records involved the date of admission and discharge. The outcomes of discharge presented as categorical variables including transferred, on-going or deceased. Likewise, the records of diagnosis presented as a categorical variable, ICD-10 for NISK, and ICD-9-CM for USSID, respectively (Fig 1B).

### Significance of disease comorbidity by time orders

Based on previous research, our disease diagnosis model used a DAG model, where nodes represent disease diagnoses and edges represent the directed time orders of the relationships between the nodes. To address the relationship between disease diagnoses, I used relative risk (RR) and quantified the co-occurrences of disease pairs (*Disease i–Disease j*) within 1 year in a patient [7]. To determine the sequential orders of disease diagnoses, I quantified the overall temporal directionality of disease co-occurrences (for *Disease i*→*Disease j*) using the mean difference in the dates of diagnoses or associated admissions for each subject [21]. I quantified the likelihood that one disease diagnosis would occur before or after another (δ_i→j_ for Disease i → Disease j) using the dates of diagnosis associated with two diseases in each individual. It begins counting date differences between when disease I was diagnosed before disease j in patient p and represent this number as d^p^_i→j_ (d^p^_i→j_ = sign (date of admission for disease j in patient p–date of admission for disease I in patient p), where sign stands for the Signum function, d^p^_i→j_ = [–1,1]). Out of re-diagnoses or re-hospitalizations for the same disease in one patient, the initial date of diagnosis for a disease was used. In addition, I only counted d ^p^_i→j_ when the length of duration between dates of admissions for disease j and disease i was less than 1 year. A value of d^p^_i→j_ > 0 indicates the following: an initial admission (diagnosis) for disease i occurred before the first onset for disease j in a patient p within one year. Then, the value of δ_i→j_ was determined by the mean value of d ^p^_i→j_ among the set of patients who were diagnosed with diseases i and j in one year. Thus, a value of δ_i→j_ > 0 indicates that over half of diagnosis for disease i occurred before the admissions for disease j by one year among the patients who were diagnosed as both of these diseases. Alternatively, a value of δ_i→_j < 0 denotes the opposite. The statistical significance of co-occurrences and temporal directionality of diseases was determined using the binomial test (Benjamin-Hochberg False Discovery Rate [FDR] <0.1) [12]. Finally, I used selected pairs of disease co-occurrences (RR >1, FDR <0.1) with a directional order of diagnoses (FDR <0.1) for further analysis.

To visualize the results, I present diseases and associated fatal outcomes as a connected path through nodes for disease (circles) that terminate in death (squares). Here, I remark that the square node means the last confirmed diagnosis without consideration of the major causality of death. For example, when patients were diagnosed with acute kidney failure and subsequently progressed to sepsis within a year, I drew a directional line (i.e., edges) between the disease nodes (disease A→B). A non-directional line was used for co-occurring deaths from each disease (disease A—death with disease A). The edge color and width represented the mean age and number of patients transferred between the disease and death nodes, respectively.

### Defining mortality trajectories and visualization

All presented DAG modeling approaches for disease diagnoses trajectories were derived from our previous studies [16–18]. In summary, I combined multiple steps of disease-to-disease trajectories by concatenating two disease progressions into three or more steps (Disease 1→2→3) based on shared patients between two pairs of disease progressions (Disease 1→2 and Disease 2→3, FDR <0.1). In case of disease diagnosis associated deaths are simply concatenated undirected edges based on the records of diagnosis outcomes from the data sources. Cytoscape [22] was employed to visualize the trajectories.

To examine the influence of age on mortality trajectories, we stratified all significant mortality trajectories from disease to death based on the mean age of the patients, representing life cycle phases of younger age groups (mean age of patients, <60 years), the elderly (mean age, >75 years), and in-between age groups (mean age, ≥60 and ≤75 years).

## Results

### Overall view of methods and data

I conducted a comprehensive analysis of administrative healthcare data associated with millions of patients, building mortality trajectories that encoded the temporally ordered progression of diseases in patients with fatal outcomes. The primary USSID study set covered 2,272,018 hospitalizations of 1,488,551 individuals from hospitals in California (Table 1). The longest interval between the first and the latest date of record for a patient extended up to 26 years (95% confidence interval [CI], 0.92–0.94 months).

**Table 1:** Data statistics of State Inpatient Dataset of California, USA (USSID)* and National Inpatient Set of Korea (NISK)**.

**Features in USSID**	**No. of record (in selected set) in USSID**
No. of admissions	19,984,041( **2,272,018)**
Data build in 2006^1^ (1980 ~ 2006)^2^	3,997,182 (128,516)
Data build in 2007^1^ (1988 ~ 2007) ^2^	4,012,774 (126,768)
Data build in 2008^1^ (1987 ~ 2008) ^2^	4,017,998 (124,632)
Data build in 2009^1^ (1988 ~ 2009) ^2^	3,985,166 (121,661)
Data build in 2010^1^ (1980 ~ 2010) ^2^	3,970,921 (1,770,441)
Grand total no of patients	10,408,641( **1,488,551)**
Data build in 2006	2,095,319 (68,237)
Data build in 2007	2,069,813 (66,611)
Data build in 2008	2,080,984 (66,048)
Data build in 2009	2,077,376 (63,691)
Data build in 2010	2,085,149 (1,223,964)
No. of diagnosis code (ICD-9-CM) ^3^	5,777
No. of diagnosis code with 3digit^3^	691
Demographic features (in selected set)	
Mean of ages in admission month	63.77 (±19.58)
Genders	Male: 691,452(46.4%), Female: 780,230(52.4%)
Outcome of admission	
Deceased patients	290,253 (19.5%)
Not deceased patients	1,334,635
**Features in NISK**	**No. of record**^**4**^ **(in selected set) in NISK**
No. of diagnoses^5^	55,291,171( **13,459,583)**
Data of 2009	19,880,745 (183,262)
Data of 2010	22,342,229 (208,124)
Data of 2011	23,031,507 (13,068,197)
Grand total no of patients	2,182,356( **763,892)**
Data of 2009	711,153 (14,629)
Data of 2010	737,854 (15,914)
Data of 2011	765,559 (733,349)
No. of diagnosis code (ICD-10)^6^	5,859 (5digit ~ 3digit)
No. of diagnosis code with 3-digit^6^	1,151
Demographic features (in selected set)	
Mean of ages in diagnose date	54.19 (±23.26)
Genders	Male: 341,788(44.7%), Female: 420,104 (55.2%)
Outcome of diagnosis	
Deceased patients (code 4)	43,545 (5.7%),
On-going patients (code1)	745,918 (97%)
Transferred patients (code 2)	21,763 (2%)
Sand backs (code 3)	13,850 (1.8%)
Others (code 5)	26,163 (3.4%)
Discharged (code 9)	444,420 (58.1%)

* Data resource of the Healthcare Cost ant Utilization Project (H.CUP) covering 97% of hospitals in US.

** Generated by random sampling covering 13% of annual hospitalization reports from HEALTH INSURANCE REVIEW & ASSESSMENT SERVICE (HIRA).

^1^Years of data generations. Merged data set covers up to ~26.1 years’ longitudinal events for a patient. It is counted by administration month. For each inpatient event, up to twenty-five diagnose codes were assigned.

^2^ Covered years of records by build year versions.

^3^Excluded injury, symptom, child-birth, pregnancy and health care service.

^4^Each data set covers 1 year’s longitudinal inpatient events for a patient.

^5^It is counted by start date of recuperations for diagnose event.

^6^Excluded injury, symptom, child-birth, pregnancy and health care service.

**Fig 2:**
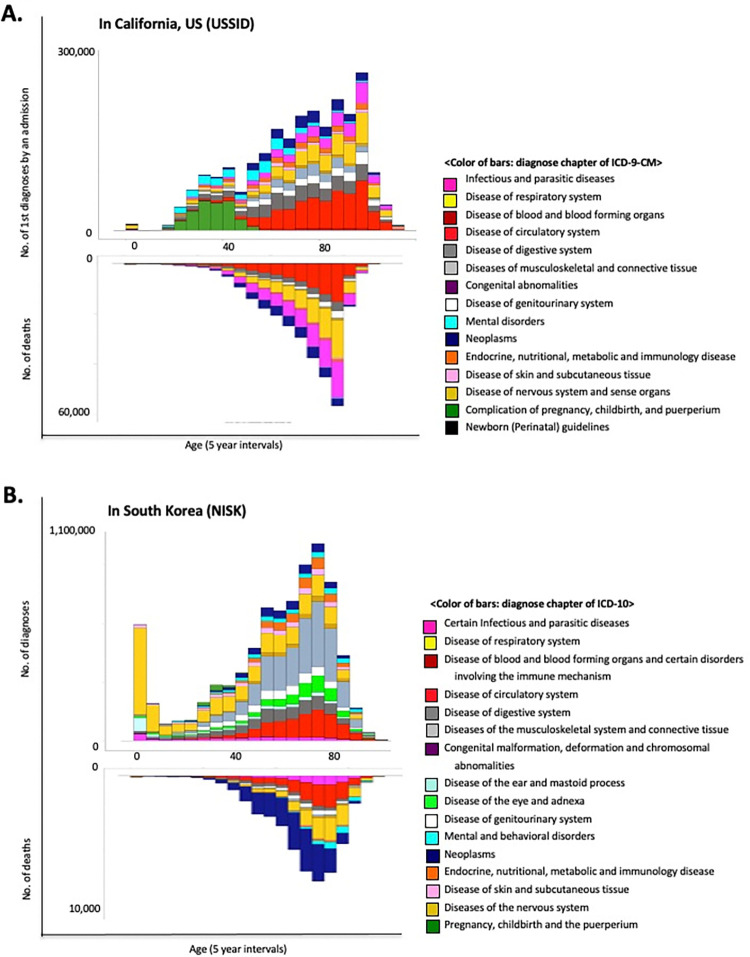
Distributions of diagnoses and associated fatal outcomes. (A) Distribution of diagnoses in the USSID by age. The bottom plot presents the distribution of the diagnoses with fatal outcomes from the USSID. (B) Distribution of diagnoses in the NISK by age. The bottom plot presents the distribution of the diagnoses with fatal outcomes among the discharges from the NISK.

An overview of the data structures (i.e., mortality) from the different sources is presented in Fig 1B and 1C. As expected, these datasets showed unmatched modalities such as distinct diagnosis codes and outpatient involvement. However, this disease trajectory modeling approach deduced the RR of disease co-occurrence and recapitulated the sequential patterns of disease diagnosis from both datasets. Interestingly, both datasets (USSID and NISK) included the outcome of diagnosis, including discharge status for deceased patients. Based on these features, I present the distributions of illness diagnoses and the assigned diagnoses confirmed to have fatal consequences across all ages in adults (Fig 2A and 2B). Owing to the collection of inpatient hospital data, USSID identifies infectious conditions, particularly sepsis, as the most frequent diagnosis associated with fatal outcomes.

Within the combined administrative healthcare records of 1.4 million patients in the US, I employed the RR of disease co-occurrence and temporal differences between diagnoses to establish the temporal order of disease progression (FDR <0.1) [7, 23]. To capture direct disease co-occurrences, I filtered for disease pairs that co-occurred within one year. I then traced patients who underwent significantly paired disease progression to determine the terminal debilitating processes (details in the Methods) and mortality trajectories through the disease toward death. For cross-sectional data from Korea (NISK), (the light blue box in Fig 1A; Table 1), the longest interval between the first and latest diagnoses in each patient is 10.4 months (95% CI, 8.13–8.15 months). Identical processes used in the US data analysis were applied to the Korean data.

### Overview of traced trajectories from initial disease diagnosis to fatal outcome

**Fig 3:**
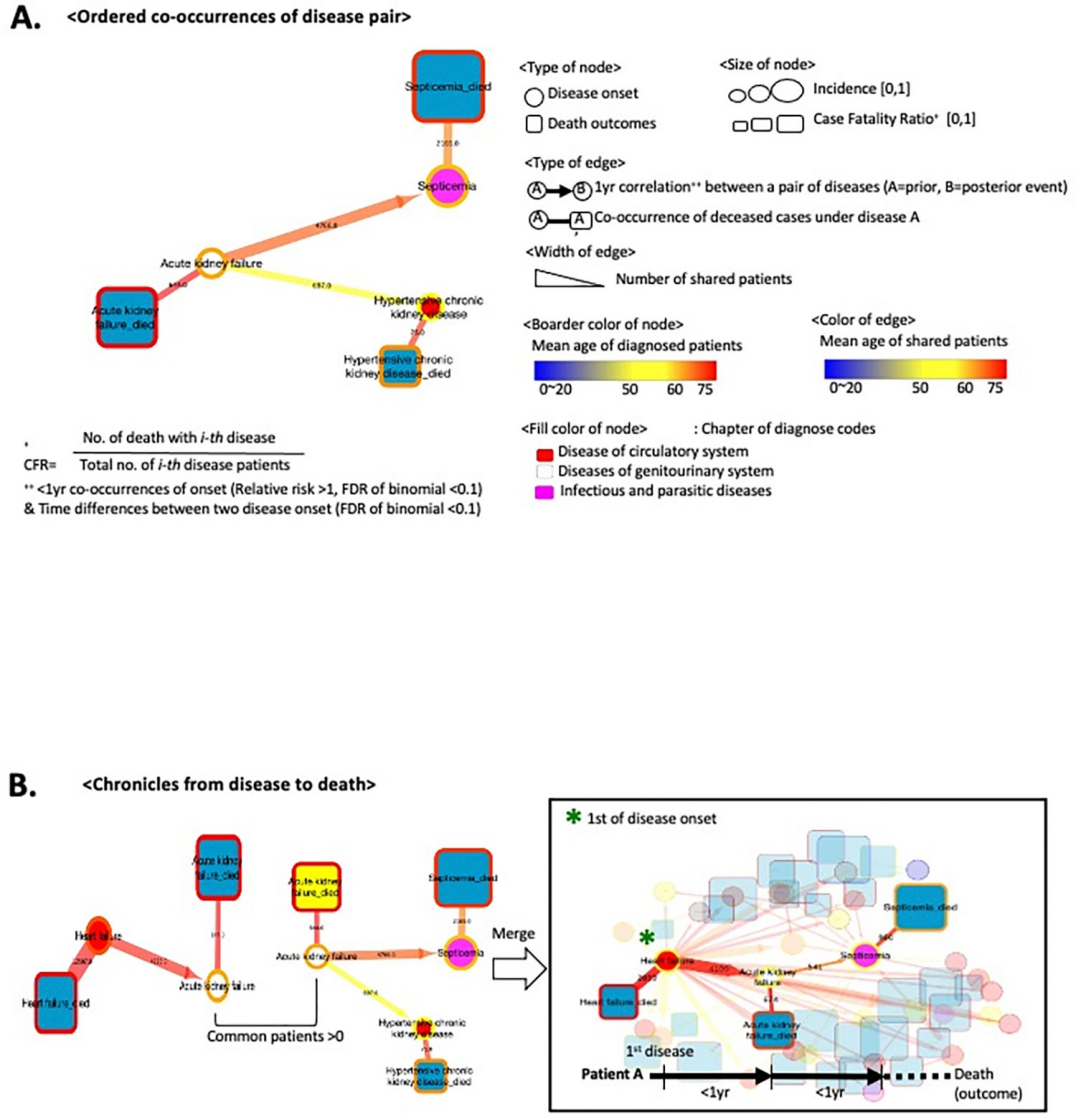
Trajectory of disease diagnosis and death. (A) Conceptual introductions to visualize timelines from disease (a circle node) to death (a square node). The linked edge (i.e. the line) between two nodes denotes significant transitions in patients within a year (FDR <0.1). (B) A simplified example of disease-to-death trajectory based on following patients from the start of the disease diagnosis.

In this study, the mortality trajectory represented the temporal order of disease progression that terminated in fatal outcomes from the initial diagnosis. All pairs of disease progression significantly co-occurred rather than being randomly distributed (FDR <0.1) [12, 23]. To visualize the paired relationship between diagnoses, I utilized directed network model consisting of nodes for diagnosis (or death outcomes) and directed edges for the identified disease progression. In Fig 3A, a circle represents a disease, such as acute kidney failure, while a square node indicates death with a disease.

To identify the mortality timelines for the majority of patients, I constructed trajectories beginning with all possible diseases using greedy search methods [12]. By drawing lines (i.e., edges) along the diagnosis history of patients among significant disease comorbidity pairs, we established the trajectory of diseases and deaths. Fig 3B illustrates a trajectory starting with heart failure. Among the 30,303 patients with heart failure, 4,109 developed acute kidney failure within a year, and 674 died from this disease. Among the surviving patients with acute kidney failure, 541 were diagnosed with septicemia, with 225 experiencing fatal outcomes.

**Fig 4:**
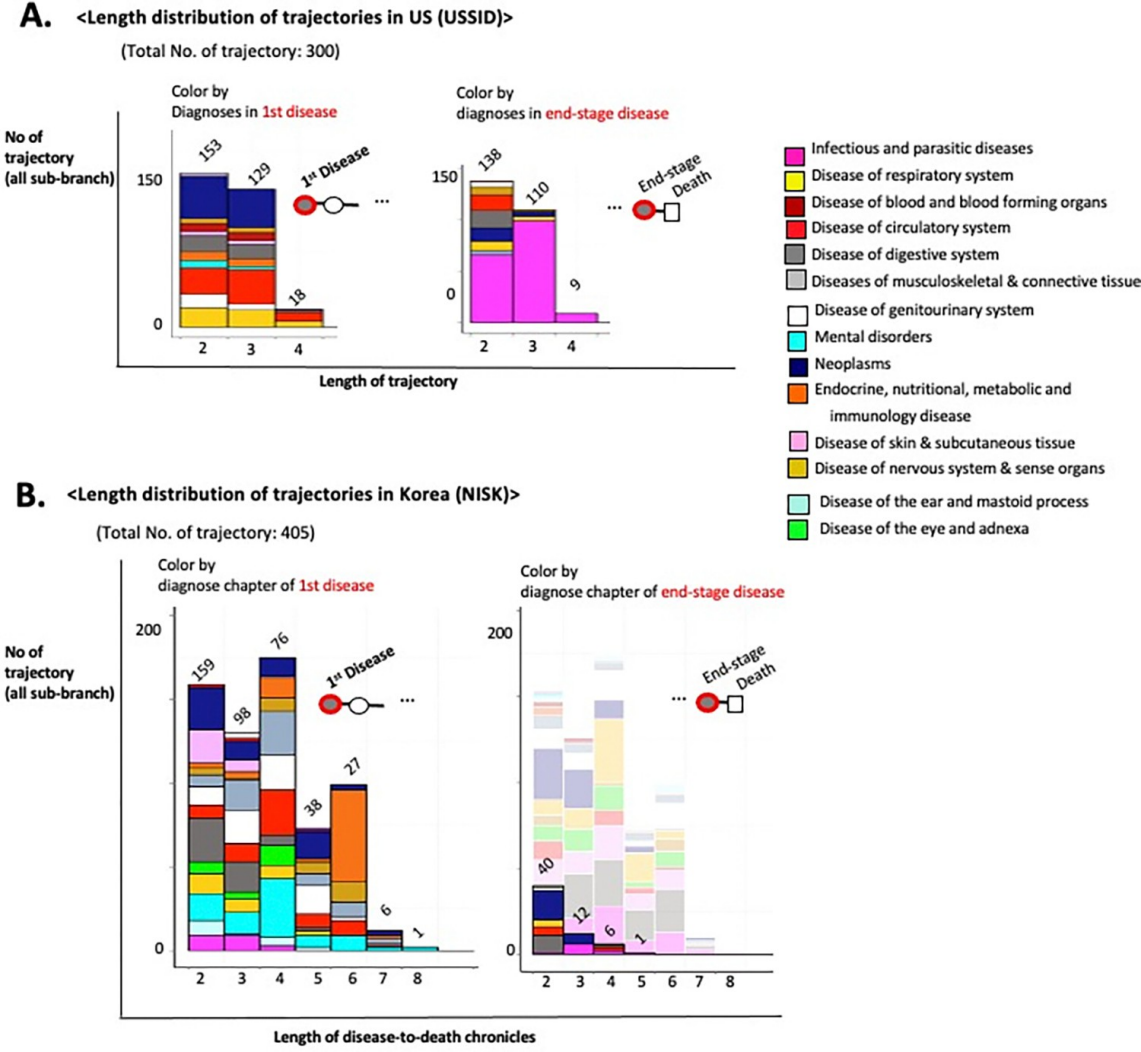
Trajectory of disease and death. (A) The length distributions of 300 identified trajectories for 59,794 deaths in the USSID. (B) The length distributions of 405 identified trajectories in the NISK. The aligned histograms of diagnosis proportion presented by initial and final diagnoses with death outcomes. Each color denotes the type of disease, as determined by ICD codes.

By systematically altering the starting disease nodes, I meticulously mapped 300 trajectories, each representing a timeline of death from the initial disease diagnosis through intermediate disease states in the US. In total, the mortality trajectories originated from 118 diseases in 311,309 patients, revealing 175,556 distinct disease-to-disease transitions and 59,794 fatal outcomes. The longest mortality trajectory was modelled for up to four steps of disease progression before the terminal death outcome, while 43 trajectories were terminated without death outcomes from the final disease progression in the trajectory (Fig 4A). The initial and interim disease nodes demonstrate heterogeneous disease types, including neoplasms (navy block) and circulatory diseases such as heart failure (red block). Nevertheless, the end stages converged into homogeneous types of diseases, such as sepsis, a disease of infection (magenta block) (Fig 4A).

Employing a similar approach to the cross-sectional Korean dataset (NISK), I identified 405 trajectories that encompassed 2,134 deaths by tracking 1,023,171 patients. Owing to the restricted timeframe of NISK (one year), a substantial proportion of trajectories (346) ended without deaths at terminal disease progression (Fig 4B). Fifty-nine non-truncated mortality trajectories followed the progression of 201,612 patients from 33 initial diagnoses to 734 deaths. Notably, 24 trajectories (54% of 59 non-truncated trajectories) reached fatal outcomes associated with cancer (Fig 4B).

These differences in the distribution of fatal outcome-associated diagnoses between the USSID and NISK mainly belong to the modality of the utilized data sources. The USSID is gathered from hospital inpatient records, whereas the NISK covers all types of healthcare insurance reviews, which indicates that there is a minimal loss of death-associated records, such as long-term care units and nursing residences. Therefore, it is essential to understand the modalities of medical records.

### Unraveling fatal outcomes using trajectories

We stratified all modeled mortality trajectories from disease to death based on the age of the patients, representing life cycle phases. The results for the in-between age group showed patterns identical to those of the overall combined group analysis.

As presented in our previous study, among all the trajectories of US data, the deadliest trajectory originates from acute myocardial infarction and ends in sepsis through a series of intermediate diseases [17] (S2A Fig). However, for younger patients (mean age, <60 years), the deadliest trajectory begins with chronic liver diseases and cirrhosis, encompassing a range of diseases, including liver abscess and sequelae, acute kidney failure, and imbalance of body fluids and acids (S2A and S2B Fig). In younger patients (mean age <60 years) within the Korean dataset, the deadliest trajectory originated from chronic viral hepatitis in 1,879 patients and resulted in the highest number of deaths (335 fatal outcomes) through the progression of cirrhosis and liver cancer (S3C Fig). Notably, this mortality trajectory among young Koreans demonstrates a well-known comorbidity pattern among East Asians [23, 24]. Therefore, although the modality of medical records affects the interpretation of the analysis results, the overall trends of health-related outcomes were also represented in the DAG-based approach.

### Unraveling fatal outcomes using pair-wise network of diagnoses

**Fig 5:**
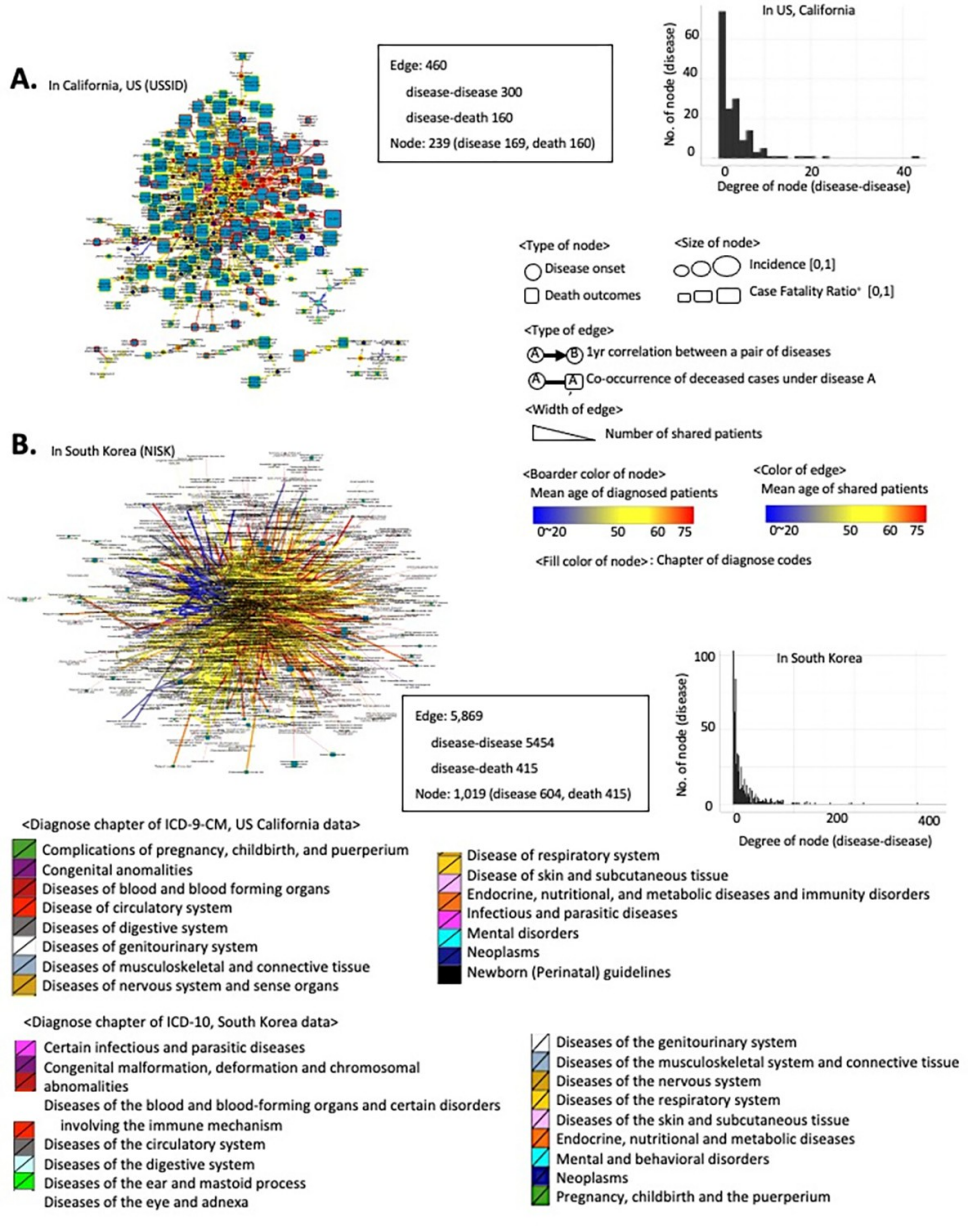
Decomposing diagnosis trajectory as a pairwise network. The traced trajectories in the USSID and NISK were decoupled into a pairwise network consisting of nodes (disease and death) and edges (directed associations). (A-B) Overview of a network of disease diagnoses and associated death outcomes in the USSID (A) and NISK (B).

Based on this confidence in the robustness of the DAG-based approach, I decomposed my diagnoses trajectories into a pairwise diagnosis-to-diagnosis network. Fig 5 presents the decomposed network of diagnosis pairs, which can be re-wired into the traced diagnoses trajectories. In the USSID, the 460 edges consisted of 300 diagnosis pairs and 160 diagnosis-death pairs (Fig 5A). In the NISK dataset, there were 5,869 edges, consisting of 5,454 diagnosis pairs and 415 diagnosis-death pairs (Fig 5B). Interestingly, most of the node degrees in NISK exceed a hundred, while those in USSID are around 60, suggesting a higher prevalence of comorbidity patterns in Korea. However, this disparity is primarily based on the modality of the utilized sources, such as the inpatient-only set from the US and the overall set from Korea.

**Fig 6:**
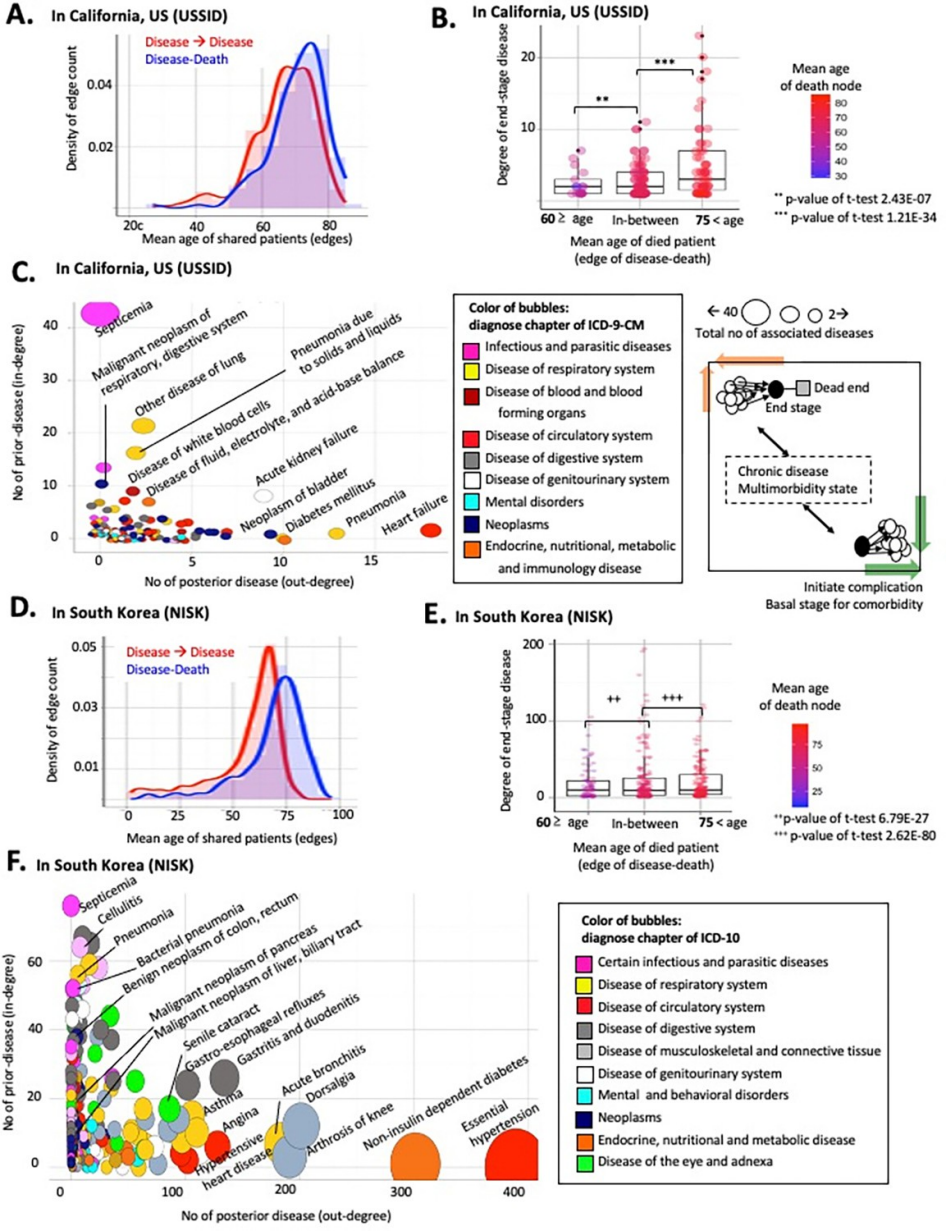
Topological features of a pairwise network of diagnosis and death. (A-B) Distributions of patient age by the type of edges including disease pairs and death-related edges in the USSID. (D-E) Distributions of patient age by the type of edges including disease pairs and death-related edges in the NISK. (C-F) Bubble plot of diagnoses nodes by the topological features. The x-axis indicates number of outlined edges, and y-axis presents degrees of in-linked edges.

To provide a more comprehensive overview of the decomposed pairwise network of diagnosis and mortality, we analyzed the age distribution of the diagnosis and fatal outcome-associated disease pairs (Fig 6A, 6B, 6D and 6E). Despite the different modalities and coverage of USSID and NISK, fatal outcomes were consistently more prevalent in elderly groups (p-value of t-test <0.05, Fig 6B and 6E).

More interestingly, the directionality of diagnosis pairs in the network highlights a number of specific disease diagnoses, including hypertension and heart failure, as common risk factors for posterior comorbid conditions, regardless of the modality of the data sources (Fig 6C and 6F). As shown in Fig 6(C) and 6(F), the x-axis quantifies the extent of the following comorbidities. Thus, the bubbles positioned towards the bottom right of the plot represent general risk diagnoses, as these initially identified conditions precede the development of multimorbidity.

**Fig 7:**
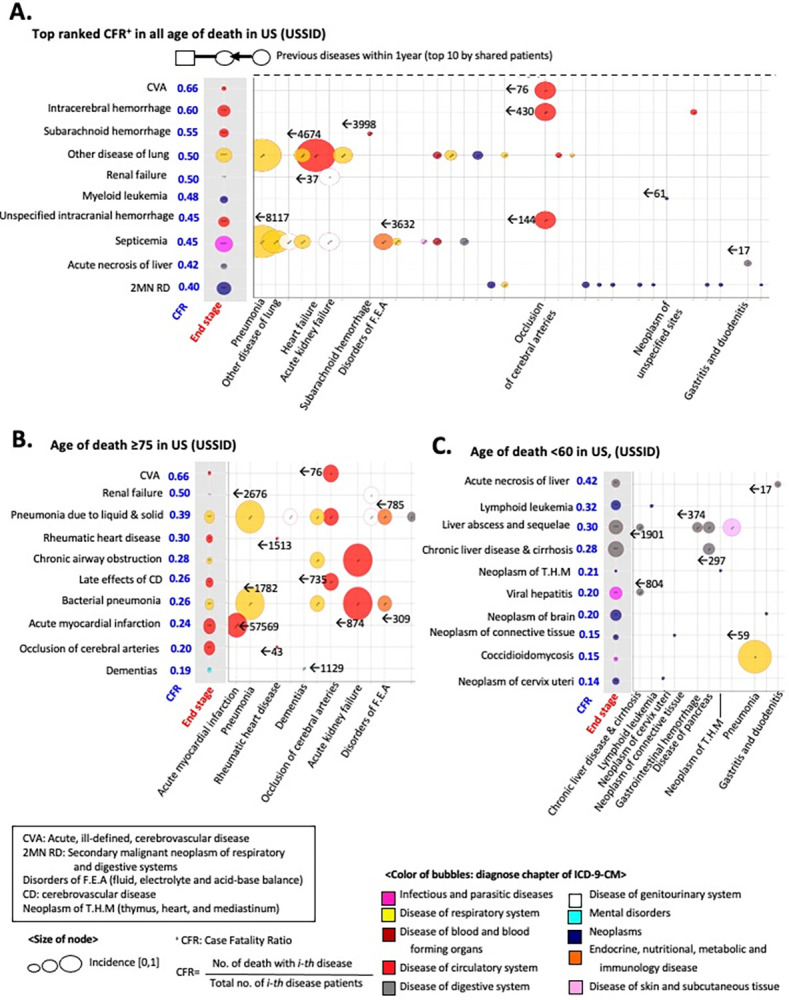
Risk factors of lethal diagnoses using the pairwise network in the USSID. The decomposed network of diagnoses identified a prior diagnoses of the diseases. (A) Detected risk factors for the top-ranked diagnosis using the Case Fatality Ratio (CFR). (B) Detected risk factors for lethal diagnoses (i.e., top-ranked CFRs) in the elderly. (C) Detected risk factors for lethal diagnoses (i.e., top-ranked CFRs) in younger patients.

**Fig 8:**
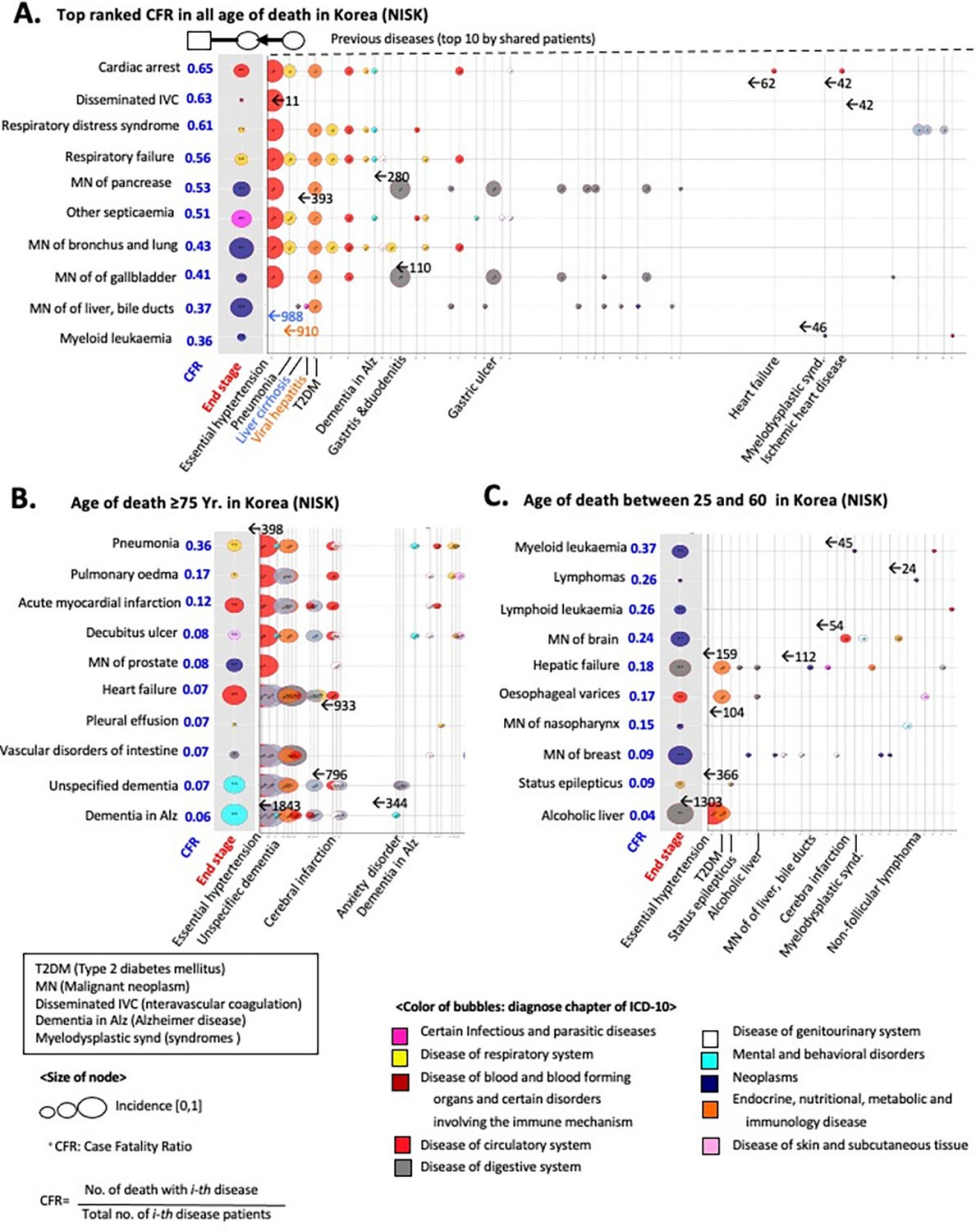
Risk factors of lethal diagnoses using the pairwise network in the NISK. (A) Detected risk factors for prioritized diagnosis using Case Fatality Ratio (CFR). (B) Detected risk factors for lethal diagnoses (i.e., strong CFRs) in the elderly. (C) Detected risk factors for lethal diagnoses (i.e., top ranked CFRs) in younger patients.

Based on this network framework, Figs 7 and 8 display the identified risk factors for lethal diagnoses with top-ranked case fatality ratios (CFRs) in the US and Korea. For example, subarachnoid hemorrhage, a notorious symptom of sudden death [25], showed the absence of a prior diagnosis (Fig 7A), whereas hypertension was a prioritized risk factor for heart failure (Fig 8B) [26]. Altogether, decoupling the diagnosis trajectory into a pairwise network catalyzes the identification of a shared risk factor for multimorbidity, calling for adequate stratification of the risk of patients.

## Discussion

In this study, I performed an extensive temporal analysis of disease-to-disease comorbidity relationships among over 600 diseases in the US, encompassing 10·4 million patients and 290,253 fatal outcomes. S4 Fig summarized entire process of conducted analysis in present study. I explored the mortality trajectories of the patients, which represented the temporal associations of significant comorbidities of diseases that lead to death. I also applied the same computational approach to the cross-sectional records of 2.1 million Korean patients. The accompanying analysis provides a comprehensive overview of health-related outcomes in different countries and modality-biased death outcomes. Therefore, interpretation of the analysis results of health-related records should be based on domain-specific knowledge.

In previous studies, we found that administrative healthcare records routinely collected for billing purposes could be repurposed for biomedical research [16–18, 27]. ICD codes and other billing information were used to classify patients and select patient cohorts for population-wide studies [28]. Here, I highlight the value of meta-data associated with administrative data, such as the temporal order of assigned ICD codes, for direct analysis. Therefore, population-wide health records that are not specifically intended for medical research are a promising source for investigating the public health of a population and risk stratification of individuals.

All analyses were conducted without sex or age stratification, as the main purpose of this study was not to address causality, but rather to identify patterns across populations. The etiological assessment of disease comorbidities and deaths, incorporating adjustments for risk factors, presents an exciting avenue for future research in precision medicine. With two distinct datasets, I experienced data alignment issues with respect to the temporal resolution of the data, quality of records, inherent noise, and other factors, which prevented a direct comparison between the US and Korean data. Thus, clinical interpretations to assess and compare the convergence and differences in trajectories between these countries must account for these differences. However, given my ability to independently replicate known epidemiological trends for patients’ life cycles in both the US and Korean populations, it can be suggested that presented method, DAG, is robust for finding strong indications of diseases that precede death.

In this study, I gained valuable insights into the utilization of large-scale population datasets. To the best of my knowledge, no previous studies have examined such diverse time-resolved spectra of mortality across diseases by repurposing the administrative healthcare records of millions of people from different countries. Although a few significant disease comorbidities have already been reported [23, 29], my approach leverages a temporal network to quantitatively model and align progression across all diseases to identify significant fatally debilitating processes. The end stages of the trajectory paths of this network converged into homogeneous states, including sepsis and pneumonia, by following distinct debilitating processes involving multiple diseases with age. In addition, the results of the decoupling of disease trajectory prioritized common risk factors including hypertension and heart failure before undergoing multimorbid conditions.

The limitations of this study are noted here. The alignment of used data sets including USSID and NISK is unsolved. Both of data sources have been gathered different purposes resulting unmatched modality. Although the open-source Observational and Medical Outcomes Partnerships (OMOP) common data model can be a solution for harmonizing digital health records, the biased data source issue remains as unsolved. For example, owing to the scope of hospital records, USSID represents a high incidence of sepsis associated deaths due to the truncation of the records of long-term care units including hospice and home-death. In addition, the analyzed data sets (i.e., USSID and NISK) have been gathered at an administrative level meaning the absence of diagnosis evidence including lab tests and pathological images. Thus, the interpretation of DAG-based analysis for each data set should be considered based on the clinical guidelines for the health professionals in each country.

I employed a systematic approach to model the trajectories from diagnosis to death, which I believe has the potential to raise physicians’ awareness and general understanding of public health determinants (S1 Data). For instance, diagnosis of ‘Mental and behavioral disorders due to use of alcohol (F10)’ showed complex associations with over thirty disease diagnoses including acute pancreatitis and seborrhoeic dermatitis in middle-aged patients indicating burden of comorbidity in public health. In addition, such systematic views of disease incidence may be useful for directing additional research to identify better biomarkers, and ultimately, drugs for the diagnoses and treatment of diseases before their fatal sequelae.

In summary, I presented an explorative and large-scale examination of mortality trajectories or dying patterns from the initial presentation of the disease across the entire disease spectrum by tracking millions of individuals’ healthcare records intended for billing. The insights gained from our study may be useful to physicians, researchers, and public health officials in changing the mortality trajectories of individual patients, thereby promoting health. For example, except the pandemic years of COVID-19, the Global Burden of Disease (GBD) study have been prioritized a cardiac disease as a long-standing leading risk across over 200 countries for decades [30, 31]. Although the previous attempts of GBD present comprehensive understanding of the burden of global health, evaluation of the method for the stratification of risk group for the initiation of clinical intervention is pending. As presented in Figs 7 and 8, suggested DAG approach allows us the identification of prior risk factors of leading lethal diagnosis, calling additional clinical intervention and prevention policy.

## Supporting information

S1 Data List of temporally correlated disease pairs in the USSID and the NISK. (XLSX)

S1 Fig Histograms of diagnosis distributions in the USSID and the NISK. (A) Distribution of diagnosis with each admission and death outcomes in the USSID by sex and ages. (B) Distribution of diagnosis with each case and death outcomes in the NISK by sex and age.
(PDF)

S2 Fig Trajectory of diagnosis with largest number of deaths in the USSID. (A) Deadliest trajectory of disease in the USSID for all ages. (B) Deadliest trajectory of disease in the USSID for young age groups (<60 years). (C) Deadliest trajectory of disease in the USSID for elders.
(PDF)

S3 Fig Trajectory of diagnosis with largest number of deaths in the NISK. (A) Deadliest trajectory of disease in the NISK for all ages. (B) Deadliest trajectory of disease in the NISK for young age groups (<60 years). (C) Deadliest trajectory of disease in the NISK for elders.
(PDF)

S4 Fig Analysis overview. *Directed Acyclic Graph modeling, details are presented in Fig 3.
(PDF)
