# Supplementary material for: Unraveling multimodality of digital health records by comparing mortality trajectories of diagnoses of diseases from over 12 million patients
Source: PLoS One. 2025 Feb 4;20(2):e0314993. doi: 10.1371/journal.pone.0314993 (PMC11793822; doi:10.1371/journal.pone.0314993)
Supplement: S1 Fig — (A) Distribution of diagnosis with each admission and death outcomes in the USSID by sex and ages. (B) Distribution of diagnosis with each case and death outcomes in the NISK by sex and age. (PDF) [file pone.0314993.s002.pdf]

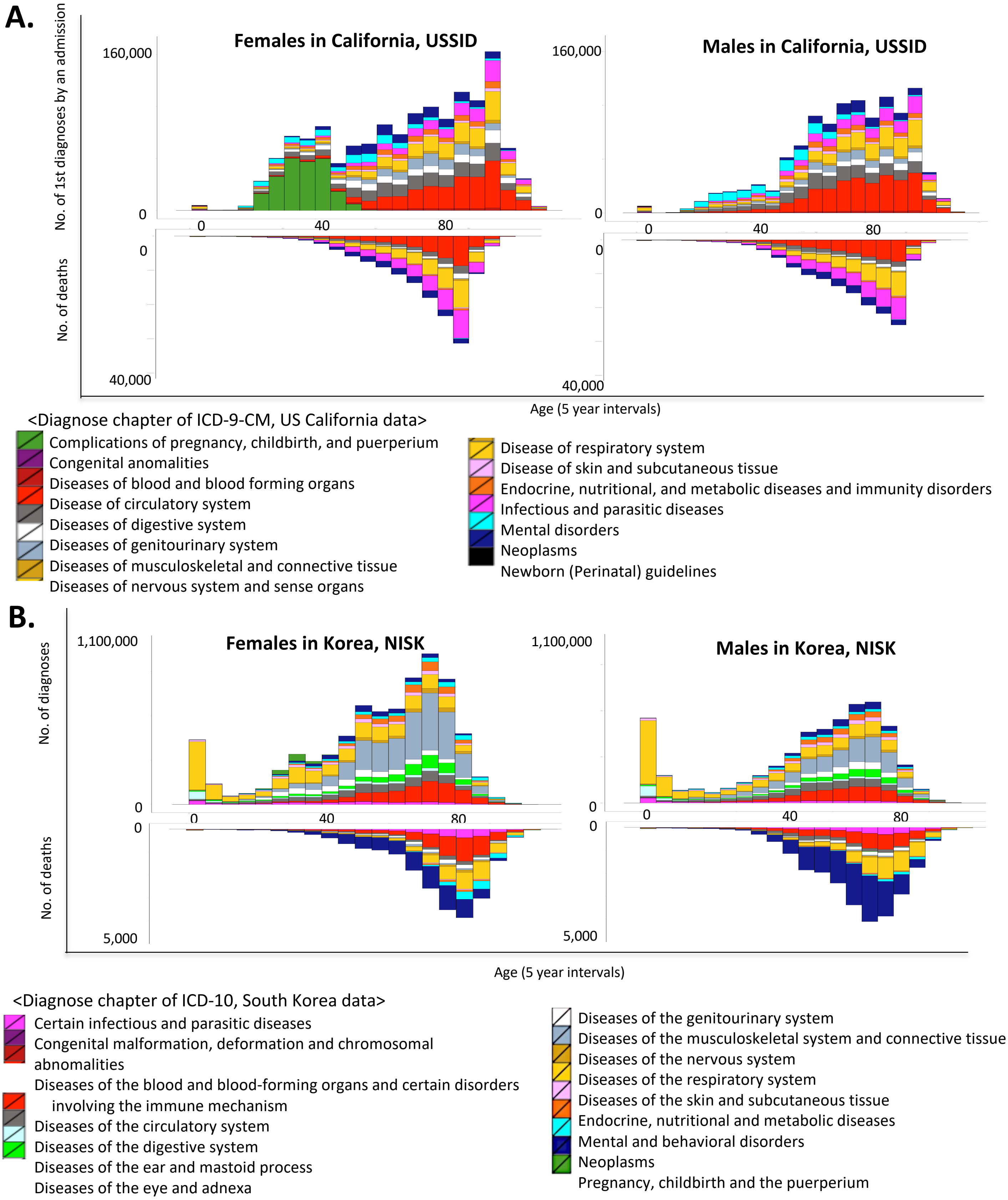

Supplemental figure 1. Histograms of diagnosis distributions in the USSID and the NISK,

(A) Distribution of diagnosis with each admission and death outcomes in the USSID by sex and ages. (B) Distribution of diagnosis with each case and death outcomes in the NISK by sex and age.
