# Supplementary material for: Unraveling multimodality of digital health records by comparing mortality trajectories of diagnoses of diseases from over 12 million patients
Source: PLoS One. 2025 Feb 4;20(2):e0314993. doi: 10.1371/journal.pone.0314993 (PMC11793822; doi:10.1371/journal.pone.0314993)
Supplement: S2 Fig — (A) Deadliest trajectory of disease in the USSID for all ages. (B) Deadliest trajectory of disease in the USSID for young age groups (<60 years). (C) Deadliest trajectory of disease in the USSID for elders. (PDF) [file pone.0314993.s003.pdf]

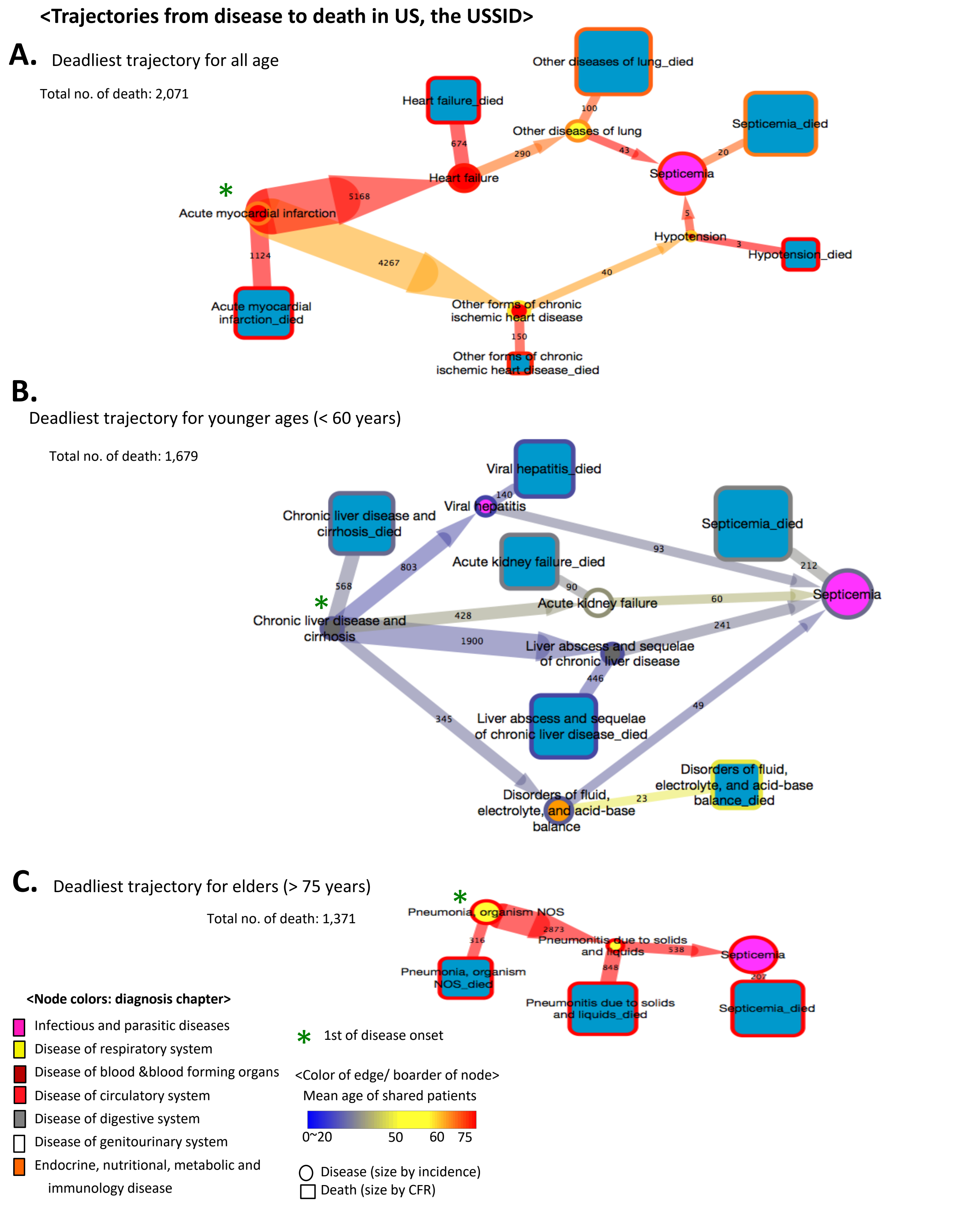

Supplemental figure 2. Trajectory of diagnosis with largest number of deaths in the USSID,
(A) Deadliest trajectory of disease in the USSID for all ages. (B) Deadliest trajectory of disease in the USSID for young age groups (<60 years). (C) Deadliest trajectory of disease in the USSID for elders
