# Supplementary material for: Unraveling multimodality of digital health records by comparing mortality trajectories of diagnoses of diseases from over 12 million patients
Source: PLoS One. 2025 Feb 4;20(2):e0314993. doi: 10.1371/journal.pone.0314993 (PMC11793822; doi:10.1371/journal.pone.0314993)
Supplement: S3 Fig — (A) Deadliest trajectory of disease in the NISK for all ages. (B) Deadliest trajectory of disease in the NISK for young age groups (<60 years). (C) Deadliest trajectory of disease in the NISK for elders. (PDF) [file pone.0314993.s004.pdf]

<Trajectories from disease to death in South Korea, the NISK>

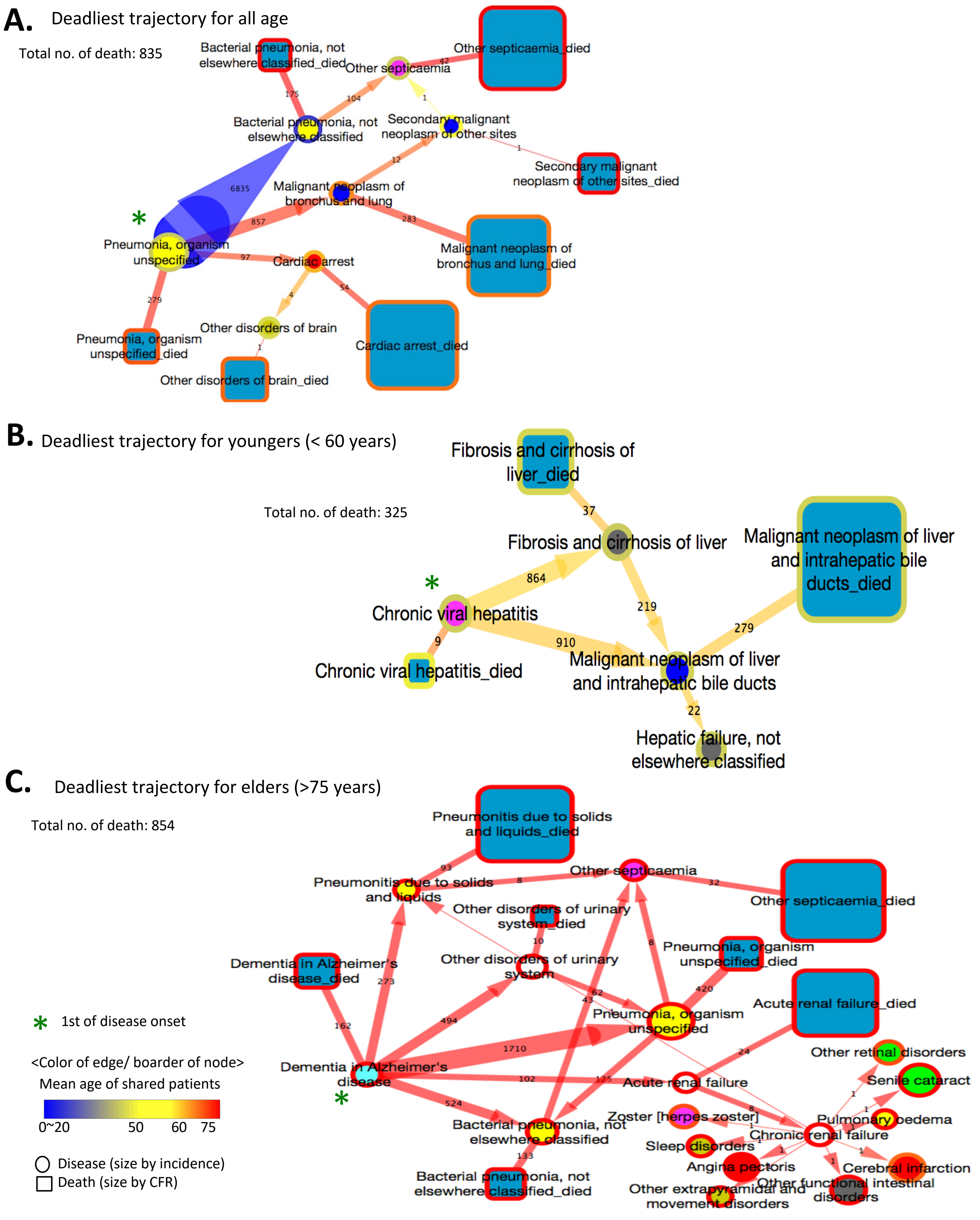

Supplemental figure 3. Trajectory of diagnosis with largest number of deaths in the NISK,
(A) Deadliest trajectory of disease in the NISK for all ages. (B) Deadliest trajectory of disease in the NISK for young age groups (<60 years). (C) Deadliest trajectory of disease in the NISK for elders
