# Supplementary material for: Unraveling multimodality of digital health records by comparing mortality trajectories of diagnoses of diseases from over 12 million patients
Source: PLoS One. 2025 Feb 4;20(2):e0314993. doi: 10.1371/journal.pone.0314993 (PMC11793822; doi:10.1371/journal.pone.0314993)
Supplement: S4 Fig — *Directed Acyclic Graph modeling, details are presented in Fig 3. (PDF) [file pone.0314993.s005.pdf]

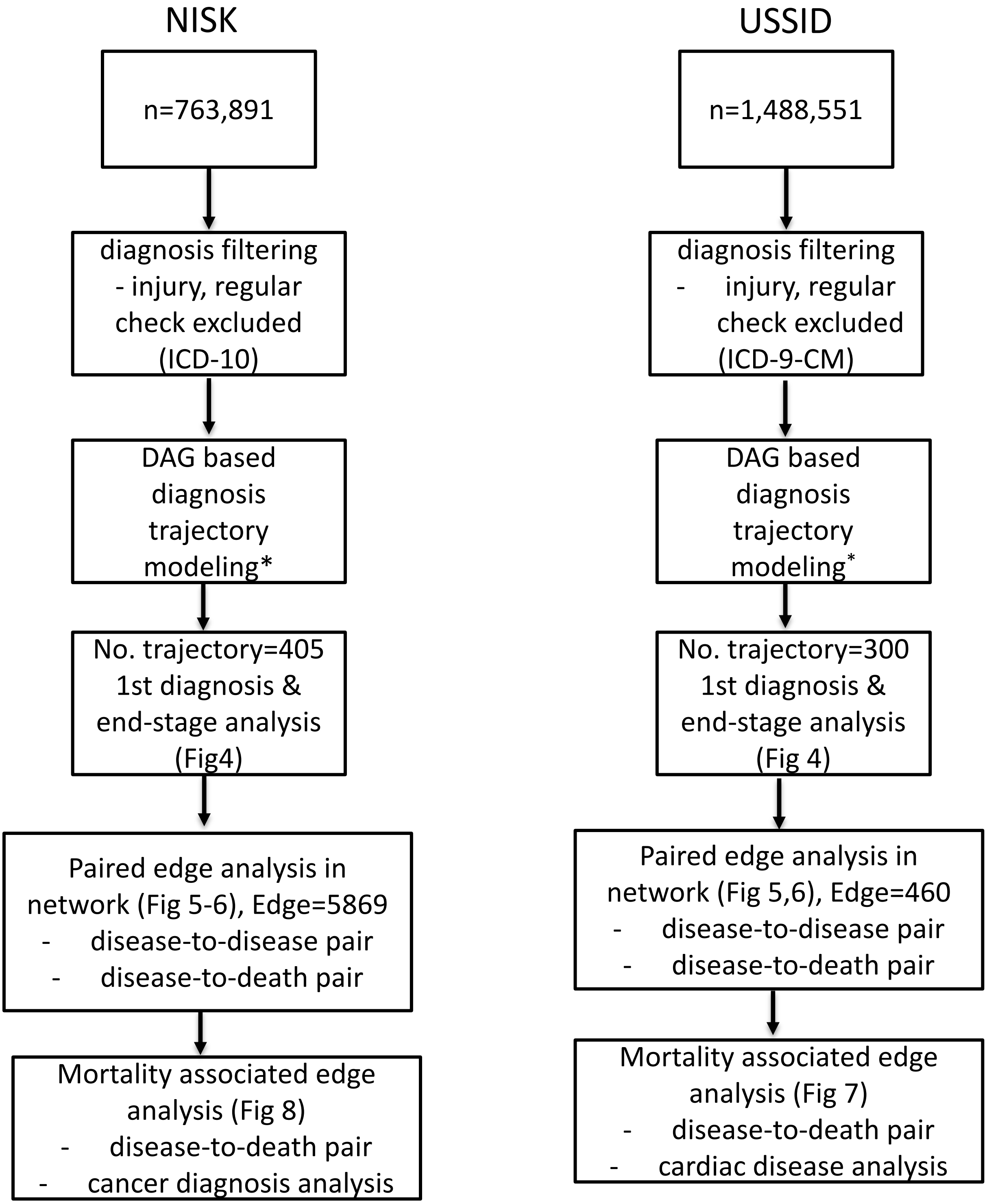

Supplemental figure 4. Analysis overview

*Directed Acyclic Graph modeling, details are presented in Figure 3
